# Altercentric Intrusions from Multiple Perspectives: Beyond Dyads

**DOI:** 10.1371/journal.pone.0114210

**Published:** 2014-12-01

**Authors:** Francesca Capozzi, Andrea Cavallo, Tiziano Furlanetto, Cristina Becchio

**Affiliations:** 1 Center for Cognitive Science, Department of Psychology, University of Torino, Torino, Italy; 2 Robotics, Brain and Cognitive Sciences, Istituto Italiano di Tecnologia, Genova, Italy; University of Udine, Italy

## Abstract

Recent findings suggest that in dyadic contexts observers rapidly and involuntarily process the visual perspective of others and cannot easily resist interference from their viewpoint. To investigate whether spontaneous perspective taking extends beyond dyads, we employed a novel visual perspective task that required participants to select between multiple competing perspectives. Participants were asked to judge their own perspective or the visual perspective of one or two avatars who either looked at the same objects or looked at different objects. Results indicate that when a single avatar was present in the room, participants processed the irrelevant perspective even when it interfered with participants’ explicit judgments about the relevant perspective. A similar interference effect was observed when two avatars looked at the same discs, but not when they looked at different discs. Indeed, when the two avatars looked at different discs, the interference from the irrelevant perspective was significantly reduced. This is the first evidence that the number and orientation of agents modulate spontaneous perspective taking in non-dyadic contexts: observers may efficiently compute another’s perspective, but in presence of more individuals holding discrepant perspectives, they may not spontaneously track multiple viewpoints. These findings are discussed in relation to the hypothesis that perspective calculation occurs in an effortless and automatic manner.

## Introduction

Recent findings suggest that, in simple visual perspective-taking tasks, one’s own and others’ visual experience influence each other [1]. On the one hand, observers are influenced by their own visual experience (egocentric intrusions) when asked to judge someone else’s perspective. On the other hand, involuntary processing of the perspective of another person can lead to intrusions of the other person’s perspective (altercentric intrusions; [1],[2]). For example, it has been demonstrated that, in level 1 visual perspective-taking tasks, when simply instructed to judge what they themselves can see, participants quickly compute the other person’s line of sight and are influenced by what she can and cannot see. This altercentric interference provide an indirect test of perspective *calculation*, suggesting that even when the task requires *selection* of a self-perspective, the other’s perspective is nevertheless computed [3],[4].

Altercentric (and egocentric) intrusions arise without instructions and are observed even when participants are given the clear opportunity to ignore the irrelevant perspective [1]. Furthermore, it has been demonstrated that participants continue to perform a computation of the irrelevant perspective during simultaneous execution of secondary executive task [3]. This indicates that perspective calculation is an efficient process that makes relatively few demands on the attentional capacity and is not disrupted (but rather increased) under conditions of cognitive load.

Taken together, these data point towards the view that observers sometimes compute another’s visual perspective in an effortless and automatic manner [3]–[5]. The boundary conditions of this phenomenon, however, are still poorly understood [6].

One limitation to our current understanding of automatic perspective computation is the focus on dyadic contexts. Altercentric intrusion effects have been reported in presence of *one* person holding a discrepant perspective. Real-world perspective-taking problems, however, frequently involve interactions with *more than one* individual. In presence of more than one person, are multiple visual perspectives spontaneously computed? Does the presence of more people cause altercentric intrusions from multiple viewpoints?

Because automatic processes use minimal attentional capacity (i.e., are efficient) [3], they do not interfere with one another [7] and can operate in parallel [8],[9]. A *strong automaticity hypothesis* predicts therefore that in the presence of more people holding different perspectives, observers should simultaneously take multiple lines of sight into account. As a consequence, multiple perspectives should be processed in parallel, causing larger altercentric interference on self-perspective judgements.

Clearly, however, there will be occasions when it is beneficial to avoid the representation of multiple perspectives, especially when they are irrelevant or otherwise distracting. Consider, for example, visiting a crowded museum and trying to focus on a specific artwork. Resisting intrusions from multiple viewpoints would be crucial to enhance the processing of the relevant stimulus, while suppressing the processing of those that are irrelevant. An alternative possibility is thus that the spontaneous processing of others’ perspective is limited to relative simple cases in which gaze cues available in the scene converge on the same objects (*partial automaticity hypothesis*; [7]). Observers may efficiently process what another person sees. In presence of more people holding discrepant perspectives, however, they may not automatically track multiple lines of sight. A *partial automaticity hypothesis* predicts therefore that the presence of two agents holding divergent perspectives should reduce (or even abolish) the altercentric intrusion effect.

### The current study

**Figure 1:**
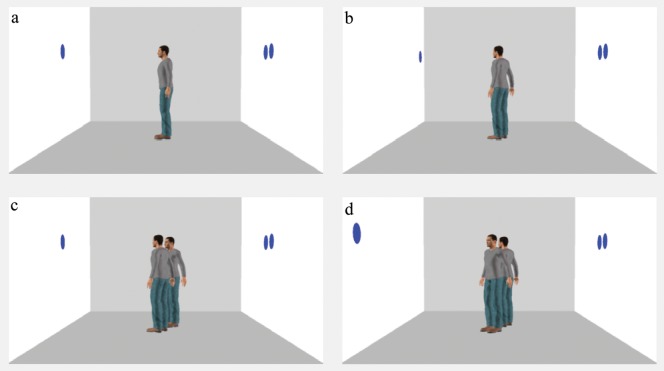
Examples of stimuli used for inconsistent trials. a) One_avatar_centered; b) One_avatar_off-centred; c) Two_avatars_centered; d) Two_avatars_off-centered. On consistent trials, the avatar(s) saw the same number of discs as the participants; the discs were thus confined to one of the walls.

To assess whether and to what extent altercentric intrusions occur from multiple viewpoints, in the current study we adapted the paradigm employed by Samson and colleagues [1] to include two avatars in the scene. Participants saw a picture of a room with one or two human avatars facing one of the walls, and with blue discs displayed on the walls. Additionally, the avatars’ bodily orientation was manipulated, so that when the scene included two agents, their gaze was directed either towards the same discs located at the centre of the wall (Figure 1c) or towards opposite sides of the wall (Figure 1d). The same layouts of discs were presented with a single avatar directing his gaze towards the centre (Figure 1a) or one side of the wall (Figure 1b).

The discs were positioned so that the participant and the avatar(s) would sometimes see the same amount of discs (consistent perspective) and sometimes a different amount of discs (the avatars being unable to see some of the discs visible to the participant; inconsistent perspective). Participants were asked to judge explicitly how many discs could be seen, either from their own perspective or from the perspective of the avatar(s), while ignoring the irrelevant perspective.

Following previous works [1]–[5], we predicted that, when the scene included a single avatar, implicit computation of the avatar’s visual perspective would interfere with self-perspective judgments. Since the avatar’s body orientation does not influence *what* the avatar can see (level 1 visual perspective taking), the size of this interference effect should be similar irrespective of the avatar’s body orientation.

When the scene included two avatars, we expected that bodily orientation would influence the altercentric intrusion effect. The direction of this influence, however, was unclear. A strong automaticity hypothesis predicts that the visual perspective of each agent in the scene is processed fast and efficiently in parallel. It follows that the inclusion of two avatars in the scene should increase the altercentric intrusion effect in comparison to when a single avatar is presented. This effect should be observed when the two avatars look at different discs, but not when they look at the same discs, since in this case the content of *what* they see (level 1 visual perspective-taking) is the same and no separate computation of their perspectives is required.

In contrast, a partial automaticity hypothesis predicts that the altercentric intrusion effect should decrease in presence of two avatars holding discrepant visual perspectives. When the two avatars look at the same discs, observers may efficiently compute what they see. When the two avatars look at opposite sides of the wall, however, their lines of sight may not be tracked. Consequently, the avatars’ perspective would no longer be available to interfere with self-perspective judgments. The distinctive finding would thus be that when the two avatars look at different discs, but not when look at the same discs, the altercentric intrusion effect is reduced in comparison to when a single avatar looks at the discs.

The manipulation of the number and orientation of the avatars in the scene was designed to probe altercentric intrusions, i.e., how computation of what the avatars see interferes participants’ judgments of their own perspective. However, an extension of the partial automaticity hypothesis would be to argue that participants do not compute their own visual perspective when judging the avatars’ perspective. Egocentrism is a recurrent characteristic in both children and adults’ perspective judgments [2]. In comparison to young children, however, adults have the executive resources necessary to resist egocentric errors, i.e., errors in the selection of the other-perspective in an explicit judgment, and may be better at correcting an automatic egocentric default interpretation when not appropriate [10],[11]. It is thus conceivable that, in presence of two avatars holding discrepant perspectives, they are less prone to intrusions from their own visual perspective. If so, a decrease in both types of intrusions may be expected. This contrasts with the prediction of the strong automaticity hypothesis, which purports that egocentric interference should remain the same whether the visual perspective of one or two agents is processed.

## Methods

### Participants

Twenty-two undergraduate students volunteered to take part in the experiment (11 females; mean age: 21.82 years, age range 19–27). All had normal or corrected-to-normal vision, were right handed, and were naïve with respect to the purpose of the study. Written informed consent was obtained from all participants. The study was performed in accordance with the ethical standards laid down in the 1991 Declaration of Helsinki and was approved by the Ethics Committee of the University of Turin.

### Stimuli

The stimuli were adapted from Samson and colleagues [1] and consisted of a picture showing a lateral view into a room with the left, the back, and the right walls visible and with blue discs displayed on one or two walls (the right or the left wall). The number of discs on each wall varied between 0 and 3. Depending on condition, one or two human male avatars, created using 3D animation software Poser 9 (Smith Micro Software), were positioned in the centre of the room, facing either the left or the right wall. Four stimulus conditions were created by varying the number and bodily orientation of the avatar(s):

*One_avatar_centered*: in which a single avatar was displayed from a full profile view (90°, left or right, with respect to the observer), so to be oriented towards the centre of the wall, as in [1] (Figure 1a);

*One_avatar_off-centered*: in which a single avatar was displayed from a three-quarter view (60° or 120°, left or right, with respect to the observer), so to be oriented towards one side of the wall (Figure 1b);

*Two_avatars_centered*: in which two avatars were displayed from a three quarter view with their face and body oriented respectively 120° and 60°, left or right, with respect to the observer, so to produce the impression that they both looked at the same discs located at the centre of the wall (Figure 1c);

*Two_avatars_off-centered*: in which two avatars were displayed from a three quarter view with their face and body oriented respectively 60° and 120°, left or right, with respect to the observer, so to produce the impression that they looked at different discs located at opposite sides of the wall (Figure 1d).

For each condition, on 50% of trials the participant and the avatar(s) saw the same number of discs (consistent perspective). On the remaining 50% of trials, the participant saw some discs that were not visible from the avatar(s)’ perspective (inconsistent perspective). In the one_avatar_centered and in the two_avatars_centered conditions the discs that were visible to the avatar(s) were displayed at the centre of the wall; in the one_avatar_off-centered and in the two_avatars_off-centered conditions the discs visible to the avatar(s) were displayed on the sides of the wall. The discs that in the inconsistent condition were visible only to the participant were always displayed at the centre of the wall (see Figure 1). The full set of stimuli is provided as Supporting Information (see Appendix S1).

### Procedure and design

The four stimulus conditions were run in separate blocks. In each condition, the position of the avatar(s) was kept constant across trials, whereas the position and the number of discs on the walls changed.

**Figure 2:**
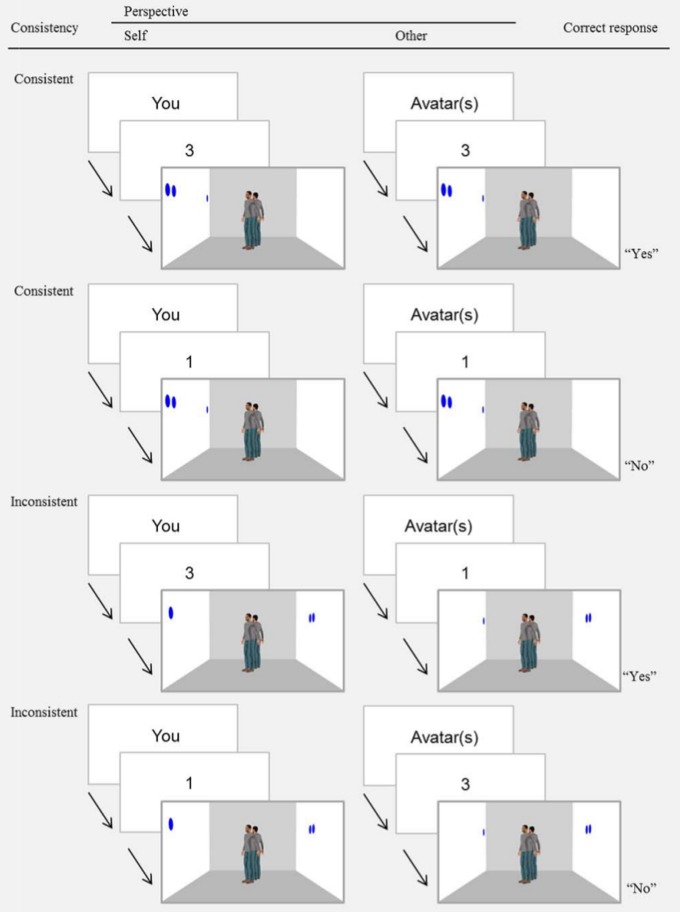
Illustration of the event sequence for matching/mismatching consistent and inconsistent trials requiring self- and other-perspective judgments in the two_avatars_off-centered condition. Please note that in Italian, English plural words (e.g., avatars) are used in the English singular form (e.g., avatar). Picture adapted from [3].

Each trial began with a fixation cross presented for 750 ms. The word “You” or “Avatar” would then appear for 750 ms, telling participants whether to take their own perspective (self-perspective, “You”) or the avatar(s)’ perspective (other-perspective; “Avatar”). Then a digit (0–3) was shown for 750 ms. Finally, the picture of the room appeared and remained on screen until a response was given or 2000 ms had elapsed. Participants were asked to indicate whether the digit matched the number of discs seen from the relevant perspective by pressing one of two keys on the keyboard. On matching trials (“yes” response), whether consistent or inconsistent, the digit corresponded to the number of discs seen from the relevant perspective (either self or other). On mismatching (“no” response) inconsistent trials, the digit specified the number of discs seen from the irrelevant perspective (i.e., the number of discs seen by the avatar(s) when the participant was asked to judge his/her own perspective, or the number of discs seen by the participant when the participant was asked to judge the avatar(s)’ perspective). On mismatching (“no” response) consistent trials, the digit specified a number of discs that did not correspond neither to the participant’s nor the avatar(s)’ perspective, and this made the response particularly easy (see Figure 2). Because of this unbalance in the construction of the mismatching responses (“no” response), following [1], we considered mismatching trials as fillers and only analysed the data of the matching (“yes” response) trials. To keep the task as similar as possible across conditions, when asked to answer from the perspective of two avatars, participants were instructed to always consider the total number of discs seen by the two avatars (i.e., the total number of discs displayed on the wall faced by the two avatars). For the two_avatars_off-centered stimulus condition, stimuli in which the two avatars saw the same number of discs were not included (see Appendix S1).

Each block contained 96 matching (“yes” response) trials, 48 self-perspective trials (24 consistent perspective trials, 24 inconsistent perspective trials) and 48 other-perspective trials (24 consistent perspective trials, 24 inconsistent perspective trials) and an equal number of mismatching (“no” response) trials (96). Each block also included 16 filler trials in which no discs were displayed on the walls (8 matching “yes” response trials, 8 mismatching “no” response trials) so that “0” would also sometimes be the correct response. Trials in each block (208 in total) were randomized. The order of the blocks was pseudo-randomized across participants to ensure that presentation order of stimulus conditions was counterbalanced. The avatar(s)’ position was kept constant across conditions. The direction of avatar(s)’ profile (left/right) was counterbalanced across participants. To acquaint participants with the visual perspective task and ensure that they correctly interpreted the stimuli, experimental blocks were preceded by a practice block. None of the participants reported difficulties in evaluating what the avatar(s) could or could not see in each condition. E-Prime V2.0 was used to control stimuli presentation and data collection (Psychology Software Tools, Inc).

### Data Analysis

For the one_avatar_centered and the two_avatars_centered conditions, discs visible from the avatar(s) point of view were displayed on the centre of the wall, whereas for the one_avatar off-centered and the two_avatars_off-centered conditions, discs visible from the avatar(s) point of view were always displayed on either the left or the right side of the wall. To control for artifacts of stimulus configuration and aid comparison between conditions, we computed for each participant and each condition an *Inconsistency ratio*, defined as the ratio of response times (RTs) for inconsistent trials and consistent trials.
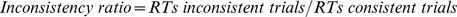



Inconsistency ratios were computed separately for the self- and the other-perspective. Values of this index greater than 1 when participants judged the avatar(s)’s perspective indicate that what participants themselves saw interfered with their judgments of the avatar(s)’s perspective (egocentric intrusion). Conversely, value of this index greater than 1 when participants judged their own perspective indicate that computation of what the avatar(s) saw interfered with participants’ judgment of their own perspective (altercentric intrusion).

Inconsistency ratios were submitted to a repeated measures analysis of variance (ANOVA) with within-subjects factors *number* (one avatar, two avatars), *orientation* (centered, off-centered) and *perspective* (self, other). Post hoc pairwise comparisons were carried out applying the Bonferroni correction for multiple comparisons when required. Additionally, one-sample t-tests (using 1 as test value) were performed on Inconsistency ratios to ascertain the effect of egocentric and altercentric intrusions for each condition. A significance threshold of p<0.05 was set for all statistical tests.

Erroneous responses (3.59% of the data) and RTs deviating more than 2 standard deviations (SD) from the mean of each experimental condition (4.32 %) were excluded from the RT analysis. RTs and mean percentage errors are reported for each condition as Supporting Information (see Table S1 and S2).

## Results

**Figure 3:**
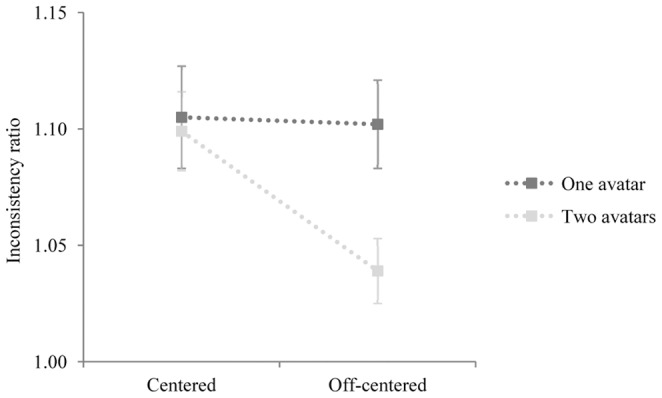
Graphical representation of the interaction *number* (one avatar, two avatars) by *orientation* (centered, off-centered) on Inconsistency Ratios. Error bars represent standard errors of the means.

The ANOVA yielded a main effect of *perspective* (F_(1,22)_ = 26.591, *p*<.0001, 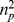 = .559), with egocentric intrusions (*M* = 1.123, *SE* = .014) producing larger interference effects than altercentric intrusions (*M* = 1.05, *SE* = .014). The main effects of *number* (F_(1,22)_ = 4.260, *p* = .052, 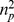 = .169) and *orientation* (F_(1,22)_ = 3.311, *p* = .083, 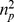 = .136) did not reach significance. The three-way interaction was also not significant (F_(1,22)_ = .755, *p* = .395, 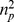 = .035), but there was a significant interaction between *number* and *orientation* (F_(1,22)_ = 5.272, *p* = .032, 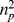 = .201), indicating that the effect of the number of avatars was different depending on the avatar(s)’ orientation. Post hoc comparisons confirmed that the two_avatars_off-centered condition (*M* = 1.039, *SE* = .014) caused smaller interference than the one_avatar_off-centered condition (*M* = 1.102, *SE* = .019) (*p* = .001). In contrast, no significant difference was found between the one_avatar_centered (*M* = 1.105, *SE* = .022) and the two_avatars_centered conditions (*M* = 1.099, *SE* = .017; *p* = .820). Further comparisons revealed that interference effects were smaller in the two_avatars_off-centered condition than in the two-avatars_centered condition (*p* = .004), whereas interference effects did not differ to each other in the one_avatar_centered and one_avatar_off-centered conditions (*p* = .916) (see Figure 3). No other interaction was significant (*Fs* <1,.454 <*ps* <.942).

Inconsistency ratios were significantly different from 1 in all stimulus conditions (other perspective: one_avatar_centered: *t*_(22)_ = 5.351, *p*<.001; one_avatar_off-centered: *t*_(22)_ = 5.184, *p*<.001; two_avatars_centered: *t*_(22)_ = 4.730, *p*<.001; two_avatars_off-centered: *t*_(22)_ = 3.304, *p* = .003; self perspective: one_avatar_centered: *t*_(22)_ = 2.126, *p* = .045, one_avatar_off-centered: *t*_(22)_ = 3.585, *p* = .002; two_avatars_centered: *t*_(22)_ = 3.162, *p* = .005), except for the two_avatars_off-centered condition on self-perspective judgments (*t*_(22)_ = .114, *p* = .910). This indicates that the altercentric intrusion effect disappeared in presence of two avatars looking at different discs.

## Discussion

Keeping track of what other people see, as well as being able to flexibly switch between self- and other-perspective, are fundamental processes to guide successfully social behavior. Prior research has focused on dyadic contexts and largely ignored the question of perspective taking in multi-agent contexts. In the present study, we sought to investigate whether spontaneous processing of perspective, as demonstrated in simple visual perspective tasks [1], extends to situations in which two people are present.

In non-dyadic contexts, people may all look at the same thing. More frequently, however, they may look at different things. Our findings indicate that the number and bodily orientation of agents modulates automatic perspective computation. When a single avatar was present in the room, participants processed the irrelevant perspective even when it interfered with participants’ explicit judgments about the relevant perspective. A similar interference effect was observed when two avatars looked at the same discs, but not when they looked at different discs. Indeed, when the two avatars looked at different discs, the interference from the irrelevant perspective was significantly reduced. This effect applied irrespective of perspective, indicating that the presence of two avatars decreased both the altercentric and egocentric interference effects.

Relative to our predictions, these results fit a *partial automaticity hypothesis* and suggest that, both when judging one’s own perspective and when judging the avatars’ perspective, observers do not process multiple viewpoints in parallel. On self-perspective judgments (but not on other-perspective judgments), the inclusion of two avatars holding different perspectives removed the intrusion effect completely, suggesting that the avatars’ irrelevant perspectives were not available to interfere with explicit perspective judgments.

One could argue that the smaller interference in the two_avatars_off-centered condition resulted from the specific layout of discs on the wall, i.e., from the fact that discs were spread on the wall rather than displayed at the centre. However, when only one avatar was present (one_avatar_off-centered), the same layout of discs did not reduce the interference effect. This rules out the possibility that the reduction of interference was an artifact of a specific stimulus configuration.

Another possibility to be considered is that the smaller interference in the two_avatars_off-centered condition resulted from slower processing of the scene on consistent trials, rather than from reduced interference from the irrelevant perspective on inconsistent trials. In the two_avatars_off-centered condition, but not in the other stimulus conditions, on consistent trials the two avatars held divergent visual perspectives. While the participant and two avatars together could see the same amount of discs, (e.g., 3), each avatar saw thus a different amount of discs (e.g., 1 vs. 2). This inconsistency between the avatars perspectives may have made perspective judgements on consistent trials as demanding as perspective judgements on inconsistent trials. To exclude this possibility, we compared RTs on consistent trials between the two_avatars_off-centered condition and the one_avatar_off-centered condition (in which the same disc configurations were used). The results clearly showed that slower processing on consistent trials was not the source of the effect. Indeed, perspective judgements on consistent trials in the two_avatars_off-centered condition (*M* = 679.38, *SE* = 33.32) were as fast as perspective judgements on consistent trials in the one_avatar_off-centered condition (*M* = 663.72 *SE* = 33.4) (*t*_(22)_ = .734, *p* = .471).

### Partial automaticity of perspective calculation

Observing two avatars looking at different discs decreased the irrelevant perspective interference in comparison to observing a single avatar. In sharp contrast, observing two avatars looking at the same discs did not reduce the effect. This indicates that the important factor in decreasing the effect was not the number of agents *per se*, but rather their orientation.

But how did the avatars’ bodily orientation impact on the implicit calculation of perspective? Gaze direction and bodily orientation provide immediate cues to the direction of social attention [12]–[14]. Observing shared attention in others has been shown to modulate action observation [15], gaze processing [16], and gaze following [17]. For example, larger gaze cueing effects have been reported when two observed individuals looked at each other and jointly gazed at an object as compared to when they have looked away from each other [17].

It is likely that similar attentional cueing effects produced by the avatars’ gaze and body orientation contributed to implicit perspective modulation in our task. When the avatars looked at the same discs, their perspectives converged on one and the same object (i.e., the same discs). Despite the avatars’ different standpoints, computation of what they saw (level 1 perspective-taking) could therefore be integrated into a shared perspective. In contrast, when the avatars looked at opposite sides of the wall, computation of what they saw required confrontation of different perspectives. It is possible that under these circumstances, the process of perspective calculation was not initiated. This would indicate that automatic calculation of the irrelevant perspective is limited to relative simple cases in which gaze cues available in the scene converge on the same object.

Alternatively, it could be envisaged that the decrease in intrusions reflected a dilution effect (e.g., [7]). Dilution effects have been reported for Stroop interference in dual conditions. For example, using a modified version of the Stroop task, it has been demonstrated that spreading visual attention by presenting a second word in the display reduces the Stroop effect [18]. Along similar lines, it could be hypothesized that the inclusion of two avatars holding divergent perspectives, by spreading visual attention, reduced the intrusion effect. If this were the case, however, we would expect the impact of the irrelevant perspective intrusion to be reduced, but not to disappear entirely. This explanation therefore, does not seem to apply to self-perspective judgments, on which the avatars’ perspective intrusion did not occur at all.

Finally, it is possible that control processes allowed observers to resists to intrusions from multiple viewpoints. Level 1 visual perspective-taking has been proposed to occur through interplay of bottom-up and top-down processes, which bias attention to the task-relevant perspective and inhibit the task-irrelevant perspective [4]. It is conceivable that, in presence of two agents holding different perspectives, inhibitory control processes intervened to suppress altercentric intrusions from multiple irrelevant perspectives [10]. On this account, the finding that the intrusion effect was abolished on self-perspective judgments, but not on other-perspective judgments, may be attributed to self-other differences in inhibitory mechanisms. Inhibiting one’s own visual perspective might be harder than inhibiting others’ perspective and this might explain why the egocentric interference effect, although reduced in size, did occur in the two_avatars_off-centered condition.

### Boundary limits of automatic perspective calculation

Cognitive efficient processing of visual perspective has been proposed to come at the cost of distinctive limits [6]. As an example of such a limitation, Apperly and Butterfill [6] and Apperly [19] suggest that Level 1 perspective taking (e.g., appreciating whether an agent sees an object that you see) may be automatic, whereas Level 2 perspective taking (e.g., appreciating how an agent sees an object from the back or from the side) may not be [20],[21]. This limitation reflects the complexity of the mental states that can be processed automatically. Our findings point towards another *boundary limit*: implicit processing of others’ perspective may be limited to tracking of convergent line of sights.

These results may seem to be at odds with extant research demonstrating that perspective calculation occurs automatically [3]. This seeming contradiction, however, can be resolved upon consideration of the multidimensional nature of automaticity [22],[23]. Current views favor a decompositional and gradual approach to automaticity and, in opposition to an all-or-none view, suggest that the presence of automaticity features such as *unintentional*, *uncontrolled*, *goal independent*, *autonomous*, *purely stimulus driven*, *unconscious*, *efficient*, and *fast* should be investigated separately [8],[24].

The present findings suggest that although level 1 perspective calculation has some features of automaticity, it is not entrusted to crude stimulus-driven control. Observers *unintentionally* [1] and *efficiently* [3] compute what others see, but do not do so in presence of more agents holding discrepant perspectives.

We speculate that this counterintuitive combination of features may guarantee the behavioral flexibility that is necessary for proper social functioning. In daily life, we are often in situations in which we observe many people attending to different objects. If we should represent what each person sees in any conceivable context, this could seriously affect the efficient processing of objects in the environment. We could miss relevant stimuli – an interesting artwork, the location of the emergency exit – simply because other people do not look at them. Invulnerability to intrusions from multiple viewpoints may maximize the adaptive value of spontaneous perspective taking and avoid ‘perspective crowding’. In this regard, an important question for future research is whether orientation cues may also influence perspective processing when the scene includes three or more individuals. Along with other social signaling cues, gaze and body orientation are used in automatic crowd analysis and have been proven to be effective tools in group detection and group tracking [25]. Subsequent experiments will be needed to determine whether spontaneous processing of ‘group perspectives’ may influence explicit perspective judgments in group and crowd situations.

## Conclusions

Spontaneous processing of the perspective of another person can lead to altercentric intrusions in dyadic contexts. Here we report evidence that the efficiency of the computation of multiple perspectives is modulated by the number and orientation of the agents in the scene. Observers implicitly and effortlessly compute what others see, but do not do so in presence of agents holding different visual perspectives. This finding demonstrates a high degree of flexibility in the ability to process others’ visual experience and suggest that multiple visual perspectives are not automatically tracked in non-dyadic contexts.

## Supporting Information

Appendix S1 **Full set of stimuli.** The stimuli marked with (*) correspond to consistent stimuli. In the one_avatar_centered, the one_avatar_off-centred, and the two_avatars_centered conditions consistent stimuli were repeated twice in order to balance the overall number of consistent and inconsistent trials. The mirror image of each of the stimuli was also presented in the experiment (balanced across subjects). Pictures adapted from [1].
(PDF)

Table S1 Reaction times data (ms) (mean ± standard error).
(PDF)

Table S2 Mean percentage errors (%) (mean ± standard error).
(PDF)
