# Supplementary material for: Altercentric Intrusions from Multiple Perspectives: Beyond Dyads
Source: PLoS One. 2014 Dec 1;9(12):e114210. doi: 10.1371/journal.pone.0114210 (PMC4250177; doi:10.1371/journal.pone.0114210)
Supplement: Appendix S1 — Full set of stimuli. The stimuli marked with (*) correspond to consistent stimuli. In the one_avatar_centered, the one_avatar_off-centred, and the two_avatars_centered conditions consistent stimuli were repeated twice in order to balance the overall number of consistent and inconsistent trials. The mirror image of each of the stimuli was also presented in the experiment (balanced across subjects). Pictures adapted from [1]. (PDF) [file pone.0114210.s001.pdf]

Appendix S1: Full set of stimuli

The stimuli marked with (*) correspond to consistent stimuli. In the one_avatar_centered, the one_avatar_off-centered, and the two_avatars_centered conditions consistent stimuli were repeated twice in order to balance the overall number of consistent and inconsistent trials.

The mirror image of each of the stimuli was also presented in the experiment (balanced across subjects).

Pictures adapted from [1].

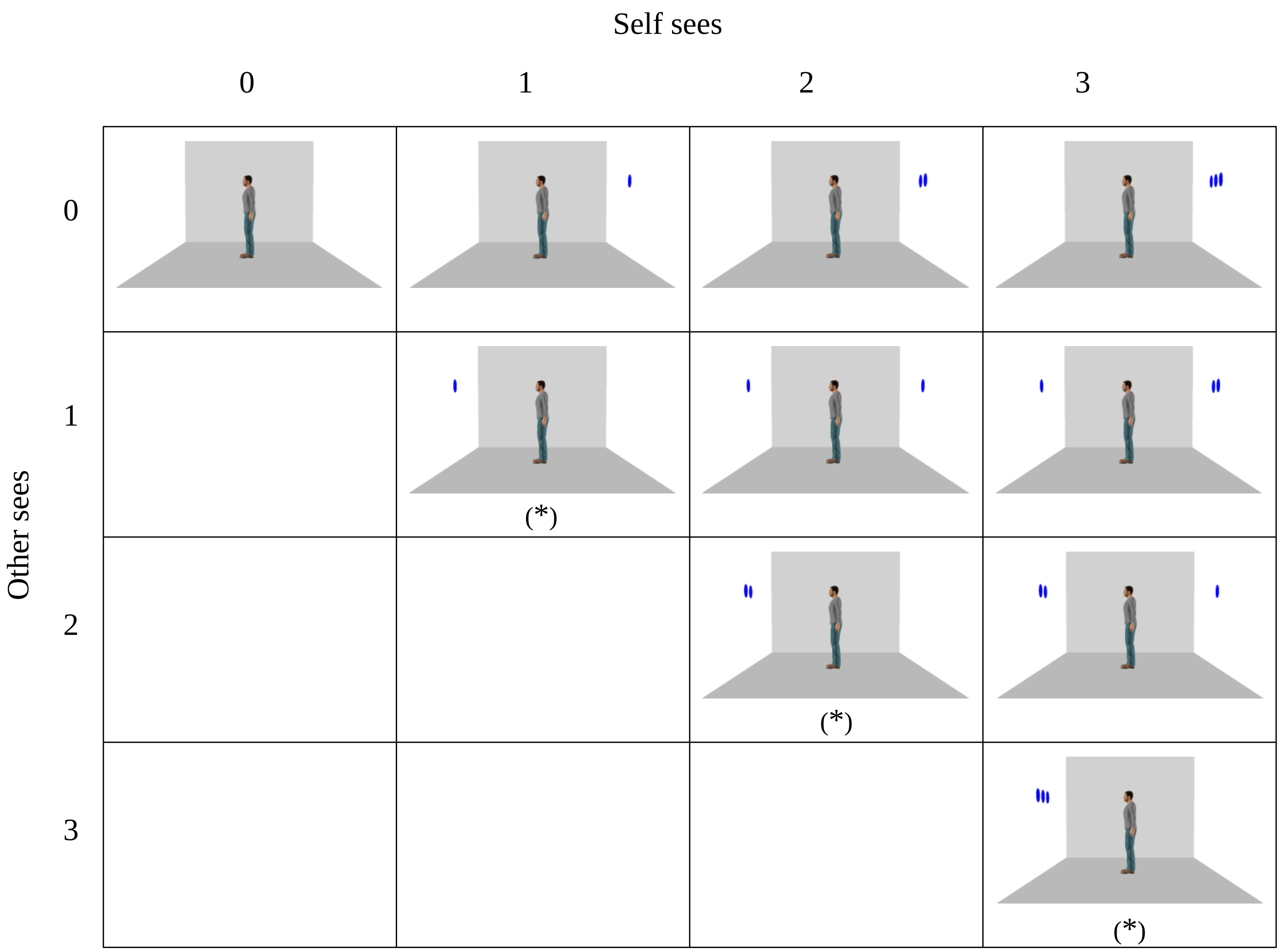

One_avatar_centered

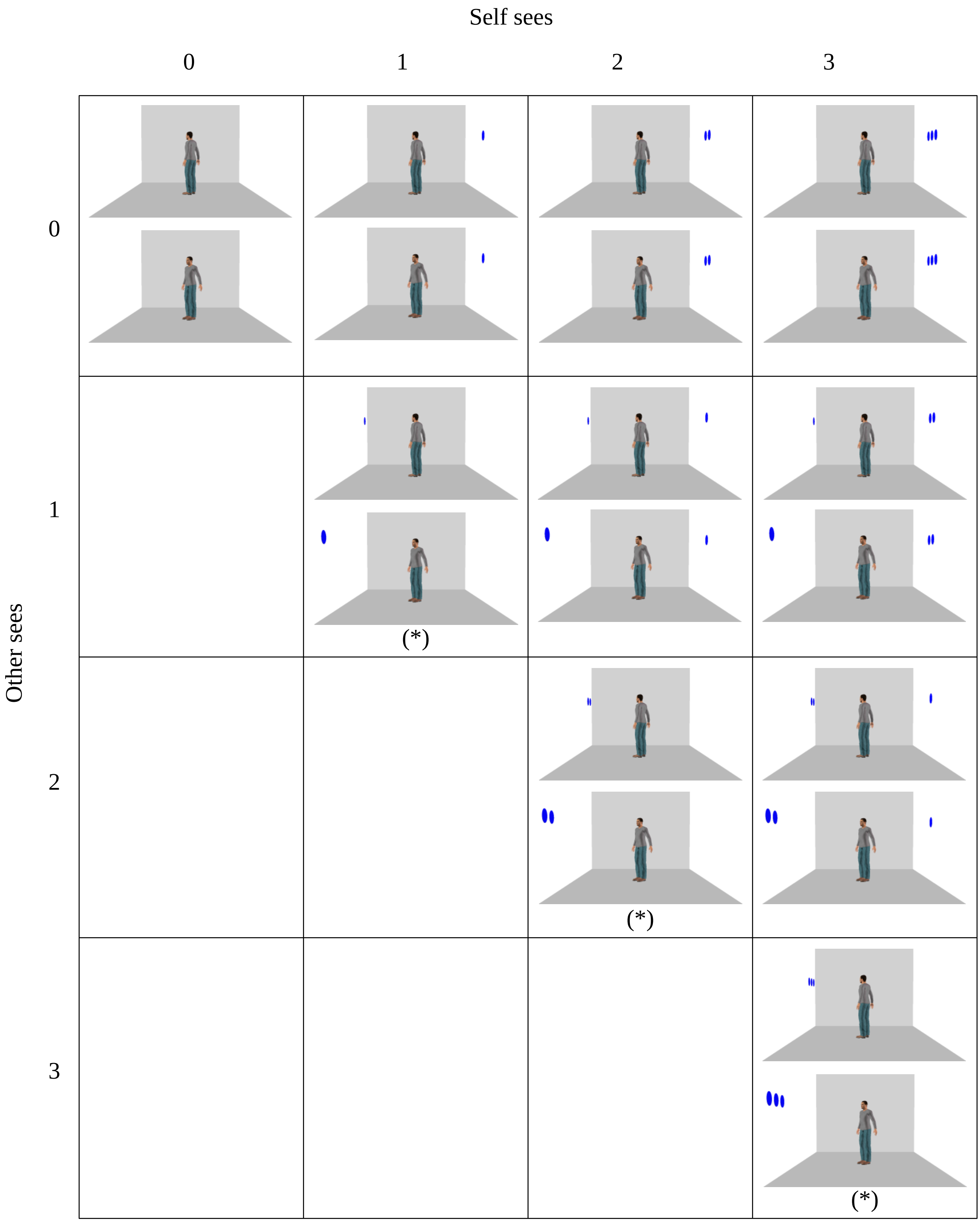

One_avatar_off-centered

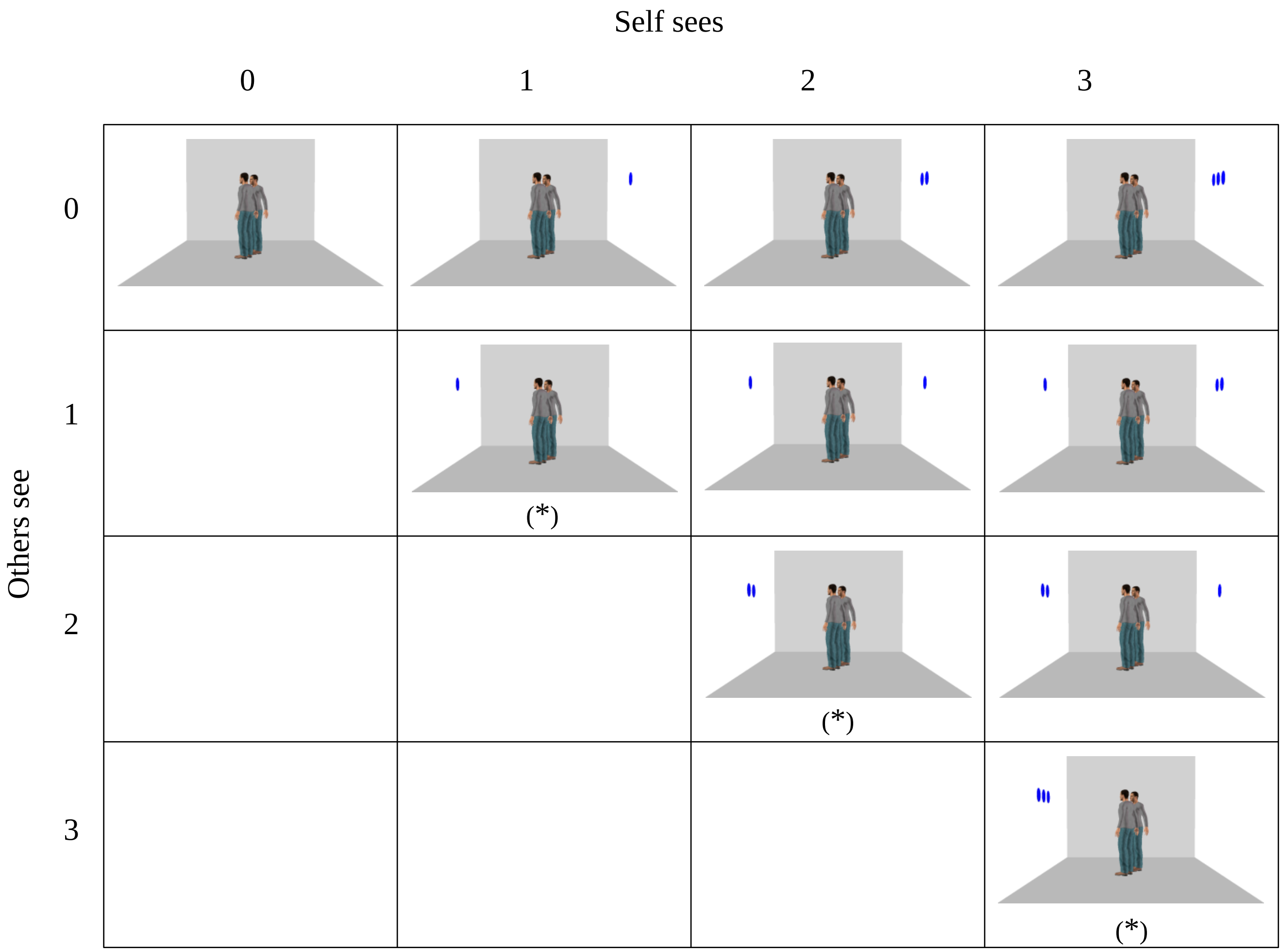

Two_avatars_centered

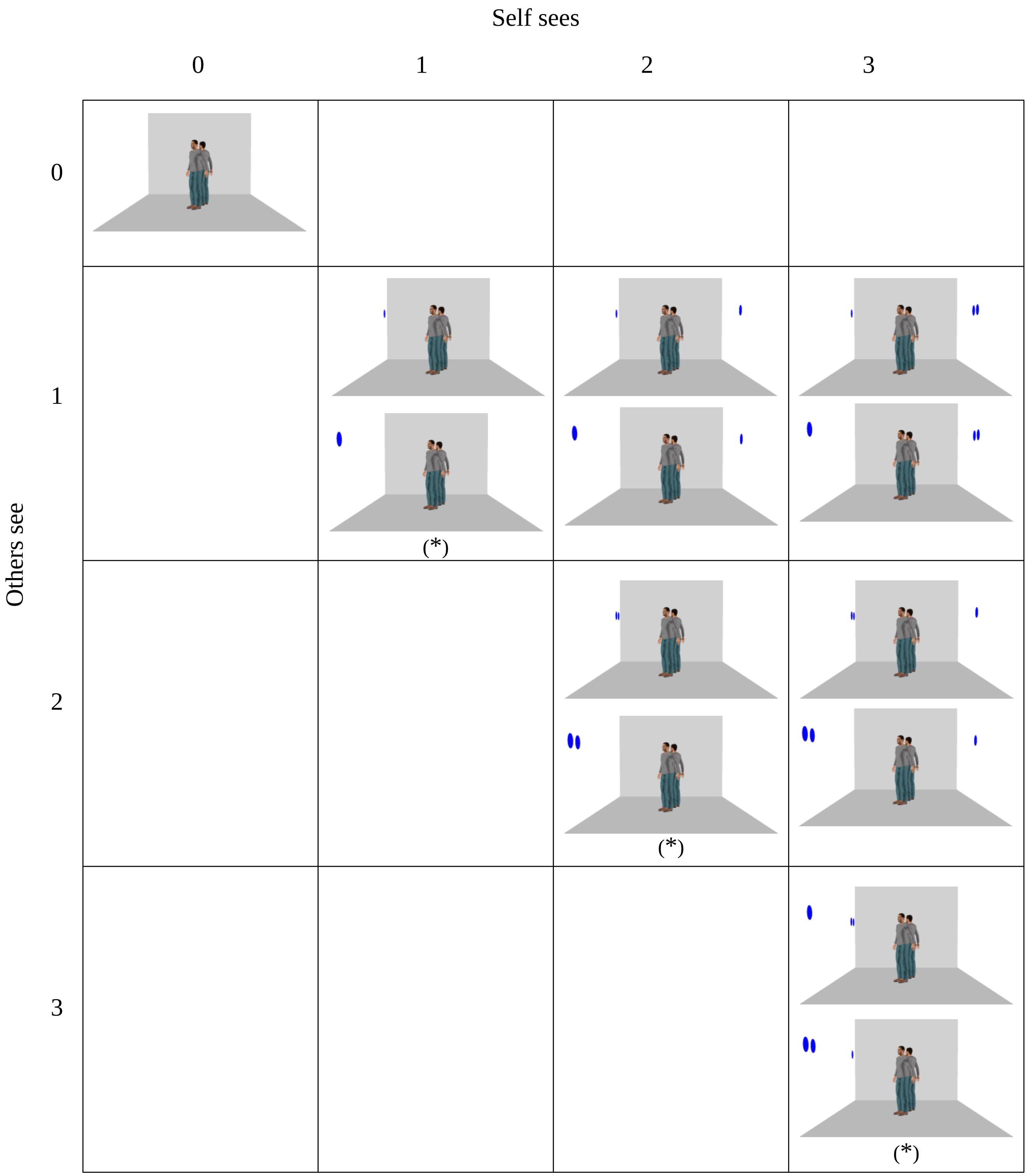

Two_avatars_off-centered
