# Supplementary material for: Altercentric Intrusions from Multiple Perspectives: Beyond Dyads
Source: PLoS One. 2014 Dec 1;9(12):e114210. doi: 10.1371/journal.pone.0114210 (PMC4250177; doi:10.1371/journal.pone.0114210)
Supplement: Table S1 — Reaction times data (ms) (mean ± standard error). (PDF) [file pone.0114210.s002.pdf]

	Self perspective		Other(s) perspective	
	Consistent	Inconsistent	Consistent	Inconsistent
One_avatar_centered	662.37 ± 34.37	692.88 ± 32.62	599.75 ± 30.21	688.40 ± 36.65
One_avatar_off-centered	689.17 ± 37.2	731.91 ± 37.39	638.28 ± 30.92	726.02 ± 37.93
Two_avatars_centered	681.83 ± 32.52	733.31 ± 37.68	638.04 ± 35.55	715.59 ± 42.87
Two_avatars_off-centered	671.56 ± 29.15	669.88 ± 27.06	687.2 ± 37.99	736.64 ± 40.11

Table S1: Reaction times data (ms) (mean ± standard error)
