# Supplementary material for: Altercentric Intrusions from Multiple Perspectives: Beyond Dyads
Source: PLoS One. 2014 Dec 1;9(12):e114210. doi: 10.1371/journal.pone.0114210 (PMC4250177; doi:10.1371/journal.pone.0114210)
Supplement: Table S2 — Mean percentage errors (%) (mean ± standard error). (PDF) [file pone.0114210.s003.pdf]

	Self perspective		Other(s) perspective	
	Consistent	Inconsistent	Consistent	Inconsistent
One_avatar_centered	1.68 ± .98	4.18 ± 1.25	.73 ± .34	6.18 ± 1.49
One_avatar_off-centered	1.5 ± .71	4.32 ± 1.19	.91 ± .45	7.73 ± 1.92
Two_avatars_centered	1.64 ± .57	5.09 ± 1.74	1.09 ± .39	6.05 ± 1.21
Two_avatars_off-centered	.95 ± .63	3.59 ± 1.15	5.09 ± 1.74	7.55 ± 1.47

Table S2: Mean percentage errors (%) (mean ± standard error)
